# Economic analyses of breast cancer control in low- and middle-income countries: a systematic review

**DOI:** 10.1186/2046-4053-2-20

**Published:** 2013-04-08

**Authors:** Sten G Zelle, Rob M Baltussen

**Affiliations:** 1 Department of Primary and Community Care, Radboud University Nijmegen Medical Centre, P.O. Box 9101 Internal Postal Code 117, 6500HB Nijmegen, the Netherlands

**Keywords:** Breast cancer control, Economic evaluation, Systematic review, Low- and middle-income countries, Cost-effectiveness, Noncommunicable diseases

## Abstract

**Background:**

To support the development of global strategies against breast cancer, this study reviews available economic evidence on breast cancer control in low- and middle-income countries (LMICs).

**Methods:**

A systematic article search was conducted through electronic scientific databases, and studies were included only if they concerned breast cancer, used original data, and originated from LMICs. Independent assessment of inclusion criteria yielded 24 studies that evaluated different kinds of screening, diagnostic, and therapeutic interventions in various age and risk groups. Studies were synthesized and appraised through the use of a checklist, designed for evaluating economic analyses.

**Results:**

The majority of these studies were of poor quality, particularly in examining costs. Studies demonstrated the economic attractiveness of breast cancer screening strategies, and of novel treatment and diagnostic interventions.

**Conclusions:**

This review shows that the evidence base to guide strategies for breast cancer control in LMICs is limited and of poor quality. The limited evidence base suggests that screening strategies may be economically attractive in LMICs – yet there is very little evidence to provide specific recommendations on screening by mammography versus clinical breast examination, the frequency of screening, or the target population. These results demonstrate the need for more economic analyses that are of better quality, cover a comprehensive set of interventions and result in clear policy recommendations.

## Background

Noncommunicable diseases (NCDs) have become increasingly important in low- and middle-income countries (LMICs). Once considered a problem only in high-income countries (HICs), more and more patients who suffer from cancers and other NCDs are now observed in LMICs [1]. This is mainly due to the ageing populations and changing lifestyles in LMICs [2]. The global importance of NCDs has recently been acknowledged through the UN Summit on NCDs, held by the UN General Assembly in September 2011. As highlighted in the summit, the most prominent cause of cancer death among women in LMICs is breast cancer, accounting for 269,000 deaths (12.7% of all cancer deaths) in 2008 [3,4].

In HICs, many efforts have been undertaken to control breast cancer, leading to various improvements in breast cancer outcomes [5,6]. Strategies for breast cancer control are geared towards early detection and early treatment, and although its benefits are still open to discussion [7-9], mammography screening has been widely implemented [10-12]. In these countries, the selection of breast cancer control strategies has often been guided by economic analyses, demonstrating the value of alternative interventions [13-16].

In contrast to the established breast cancer control strategies in HICs, breast cancer is often neglected in LMICs and control strategies lack evidence-based information [17-20]. Policy-makers in LMICs cannot adopt similar breast cancer control strategies as implemented in HICs because most LMICs rely on much smaller budgets, and both the costs and effectiveness of control strategies are highly dependent on the population characteristics and the functioning of the health system [11,20,21].

Against this background, the present review provides an inventory of economic analyses of breast cancer control in LMICs. The paper’s objectives are to present the available economic evidence from LMICs and to assess the methodological quality of the analyses. This research could improve the evidence base on cost-effective breast cancer interventions and could strengthen breast cancer control policy in LMICs.

## Methods

### Search strategy and selection criteria

In this review, we analyzed publications from the MEDLINE index using PubMed, the Web of Science, Scopus, and Google Scholar. We searched the literature using the keyword ‘breast cancer’, combined with the keywords: ‘developing countries’, ‘Asia’, ‘USSR’, ‘Middle-East’, ‘Eastern Europe’, ‘West-Indies’, ‘China’, ‘Russia’, ‘India’, ‘Africa’, or ‘limited resource’, or combined with: ‘cost-benefit’, ‘cost-effectiveness’, ‘costing’, or ‘cost analysis’. Additionally, we searched these indexes using ‘breast neoplasms’, ‘developing countries,’ and ‘economics’ in MeSH terms. Our search took place in January 2013, and was limited to publications in English. Studies were included only if they concerned breast cancer and originated from LMICs as listed by The World Bank [22].

**Figure 1:**
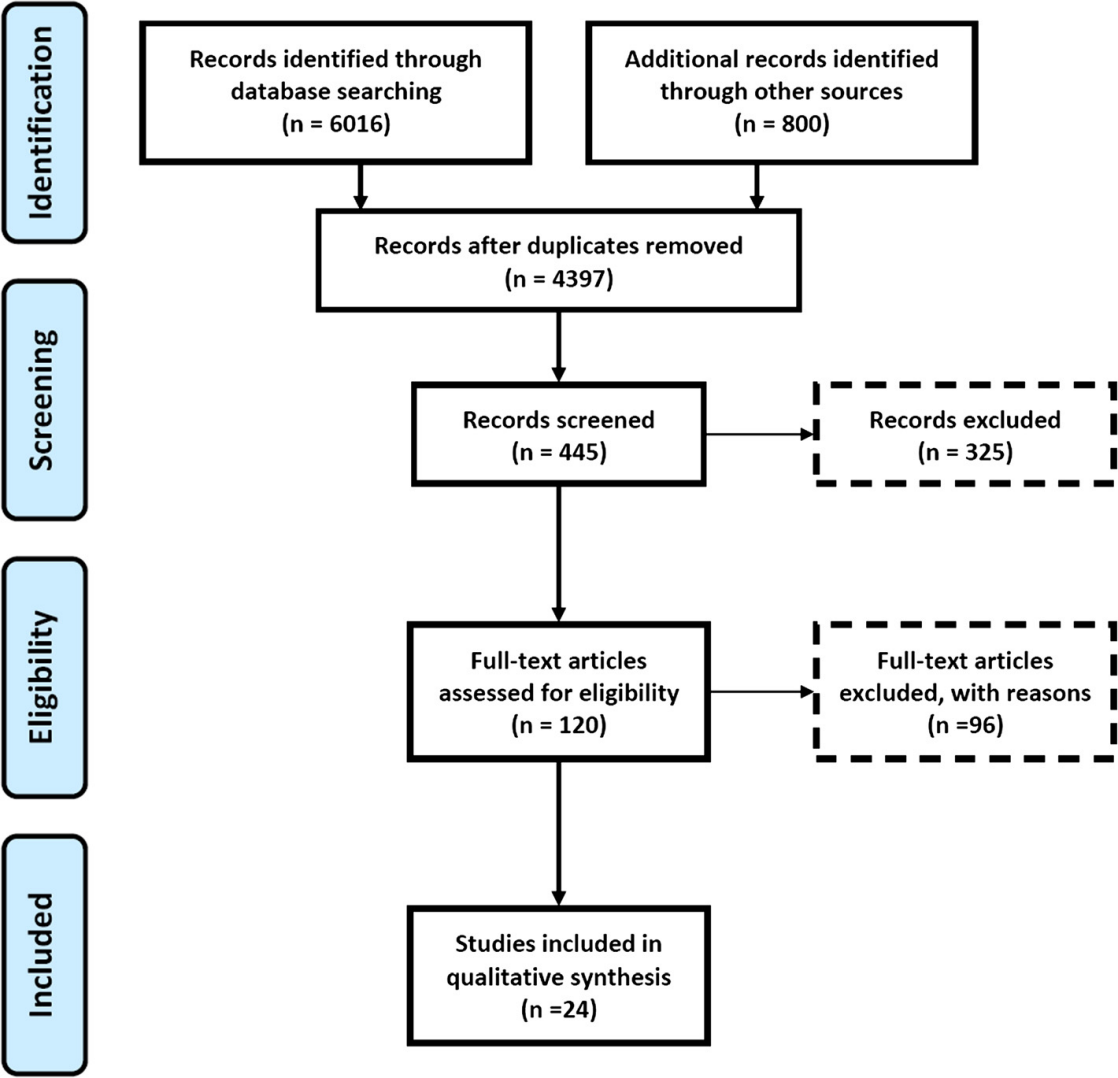
Prisma statement 1: Prisma 2009 flow diagram.

The selection process is shown in Figure 1. In step 1, articles found by our search in the various indexes were merged in a database, which was then corrected for duplications (in Google Scholar, because of the large number of articles founds, we screened titles until the point that we did not find any further relevant title among the last 500 screened titles; in total, we screened 800 titles in this database). In step 2 we screened the titles of these articles, in step 3 the abstracts and in step 4 the remaining articles were read completely. We excluded publications for which no full-text article versions were available, or those not published in English. Furthermore, we excluded articles that only mentioned costs or cost-effectiveness without presenting original data.

### Study characteristics

We documented the following characteristics from the reviewed articles: country or region, base year of cost data, study population, and breast cancer stage(s) considered. The stage was categorized as stage I to IV according to the American Joint Committee on Cancer [23].

We documented the following methodological characteristics: type of economic evaluation –cost analysis or cost of illness analysis, separately reported costs and effects, cost-effectiveness analysis, cost–benefit analysis, and cost–utility analysis; study design – experimental, observational (cohort, case control, or cross-sectional), model based, and other designs; study perspective – non-healthcare perspective (for example, productivity loss, travel costs, co-payments), healthcare perspective (for example, hospital administration costs, treatment costs), and societal perspective including non-healthcare and healthcare costs; time horizon; and outcome measure for effectiveness (disability-adjusted life years, quality-adjusted life years, life years saved, lives saved, and intermediate outcome measures).

The following qualitative characteristics were documented: sources for estimation of effectiveness, sources for estimation of resource utilization, discount rates used, sensitivity analysis for assumptions, and reported incremental analysis. We classified sources for estimation of effectiveness and resource utilization by primary data collection (for example, patients, questionnaires), secondary data collection (for example, records), literature based, expert opinion, and other. We also noted whether discount rates were used on costs, effects, both costs and effects, or not at all.

We also registered the study objective, the evaluated interventions, and the main study conclusions for each reviewed article.

### Study evaluation

We used an established checklist by Drummond and Jefferson to judge the quality of the economic evaluations [24,25]. A three-point response scale was added, similar to Gerard and colleagues [25], to more specifically grade the quality of each item on the checklist. Scores on this scale ranged from 0 (not considered), to 1 (partially considered) to 2 (fully considered). A few adjustments to the checklist by Drummond and Jefferson were necessary to create a more responsive scoring system for our particular set of economic studies. We removed those items that were not applicable to any of the reviewed studies (for example, on productivity changes), and combined some items that were otherwise putting too much emphasis to certain domains in the overall score (for example, on health state valuations and discount rates). The adapted checklist is provided in Table 1. We summed up all scores, and compared this with the maximum attainable score to calculate the mean quality score of a study (as a percentage of the maximum attainable score). We accounted for items that were not relevant to the study under scrutiny (for example, studies that studied costs and effects in a single year were not criticized for not applying any discount rate in the analyses).

**Table 1:** Checklist for quality of economic evaluations

**Item**	**Fully**	**Partial**	**Not at all**	**Not appropriate**
Original checklist	2 points	1 point	0 points	NA
Study design				
1. The research question is stated	□	□	□	□
2. The economic importance of the research question is stated	□	□	□	□
3. The viewpoint(s) of the analysis are clearly stated and justified (relating to a particular decision-making context)	□	□	□	□
4. The rationale(s) for choosing the alternative programs or interventions which are compared is stated	□	□	□	□
5. The alternatives being compared are clearly described	□	□	□	□
6. All relevant alternatives are included	□	□	□	□
7. The choice of economic evaluation is justified in relation to the questions addressed	□	□	□	□
Effectiveness estimation				
8. The primary outcome measure for the economic evaluation is clearly stated	□	□	□	□
9. The source(s) of effectiveness estimates used is clearly stated	□	□	□	□
10. Details of the design and results of the effectiveness study are given (if based on a single study)	□	□	□	□
11. Details of the methods of synthesis or meta-analysis of estimates are given (if based on multiple studies)	□	□	□	□
12. Data and methods used to value health states and other benefits are stated and justified.	□	□	□	□
Cost estimation				
14. Indirect non-healthcare costs are included or discussed	□	□	□	□
15. Quantities of resources are reported separately from their unit costs	□	□	□	□
16. Methods for the estimation of quantities and unit costs are described and justified.	□	□	□	□
17. Details of currency of price adjustments for inflation or currency conversion are given	□	□	□	□
Analysis				
18. Time horizon of costs and benefits are stated	□	□	□	□
18. Details of any model used are given	□	□	□	□
19. The choice of model used and the key parameters on which it is based are justified	□	□	□	□
20. The discount rate(s) is stated	□	□	□	□
21. The choice of rate(s) is justified	□	□	□	□
22. Details of statistical tests and confidence intervals are given for stochastic data	□	□	□	□
23. Sensitivity analysis is performed: 2) Probabilistic (bootstrap/Monte Carlo) 1) Deterministic (one way /multiple way)	□	□	□	□
24. The choice of variables in sensitivity analysis and the range over which these variables are varied is justified	□	□	□	□
25. Incremental analysis is performed and reported	□	□	□	□
Interpretation of results	□	□	□	□
26. Major outcomes are presented in a disaggregated as well as aggregated form	□	□	□	□
27. The answer to the study question is given	□	□	□	□
28. Relevant alternatives are compared	□	□	□	□
29. Conclusions follow from the data reported	□	□	□	□
30. Conclusions are accompanied by the appropriate caveats such as generalizability, equity, feasibility, and implementation	□	□	□	□

This checklist was adapted from Drummond and Jefferson [24].

Two reviewers (SGZ and RMB) evaluated each publication for conformance with this checklist, and consensus was reached when scores differed. We followed PRISMA guidelines for reporting this systematic review.

## Results

### Search results

The stepwise selection of articles by our selection criteria is presented in Figure 1. Our search strategy resulted in a total of 6,816 studies: 679 studies from PubMed, 328 studies from Web of Science, 5,009 studies from Scopus, and 800 from Google Scholar, respectively. In step 1, by merging the results of all individual search strategies and excluding duplication, the total number of hits was reduced to almost 4,400. Upon screening of titles (step 2), abstracts (step 3) and full texts (step 4), we eventually identified 24 articles that met our inclusion criteria.

### Study characteristics

Table 2 describes the baseline characteristics of the 24 included studies. We found eight studies from Asia, most concerning China, India and Iran. Five studies were on a global or sub-regional level, while there were five studies from Africa, three from Europe and three from Latin America. A total of 10 studies evaluated breast cancer screening in combination with treatment (*n* = 10), assessing mammography screening (*n* = 9), clinical breast examination (CBE) (*n* = 3), magnetic resonance imaging (*n* = 1), ultrasound (*n* = 1), biopsy (*n* = 1), elasticity imaging (*n* = 1), and tactile imaging (*n* = 1), respectively [26-36]. These studies evaluated a variety of age groups and screening frequencies (Table 3). One study reported on a mass-media intervention to improve the early detection of breast cancer in Ghana [35]. Seven studies evaluated only treatment interventions including drug therapy (*n* = 4), oophorectomy (*n* = 1), radiotherapy (*n* = 1), and treatment in general (*n* = 1) [37-42]. Other studies examined the costs of diagnostic interventions (*n* = 3) or did not consider a specific intervention (*n* = 2) [43-48].

**Table 2:** **Characteristics of reviewed studies**, **ordered by base year of cost data**

**Authors**	**Region** / **country**	**Base year of cost data**	**Study population**	**Breast cancer stage considered**	**Economic evaluation type**	**Study design**	**Perspective**	**Time horizon**	**Effectiveness outcome measure**	**Sources for estimation of effectiveness**	**Sources for estimation of resource utilization**	**Discount rates used**	**Sensitivity analysis for assumptions presented**	**Incremental analysis reported**
Groot and colleagues, 2006 [28]	World sub-regions	2000	Female population at risk, in AfrE, AmroA, SearD	All	Cost-effectiveness analysis	Model based	Healthcare	100 years	DALYs	Literature based	Secondary data collection	On both costs and effects	Yes	Yes
Okonkwo and colleagues, 2008 [30]	India	2001	Female population at risk	All	Cost-effectiveness analysis	Model based	Healthcare	25 years	Life years saved	Secondary data collection	Secondary data collection	On both costs and effects	Yes	Yes
Munshi, 2009 [41]	Worldwide	Varying from 2002 to 2007	Breast cancer patients in general	All	Report on costs and effects separately	Other	Healthcare	NA	Intermediate outcome measures	Literature based	Literature	NA	NA	NA
Sarvazyan and colleagues, 2008 [32]	Worldwide	Varying from 2003 to 2007	Female population at risk	All	Cost-effectiveness analysis	Other	Not stated	1 year	Life years saved	Literature based	Literature	NA	Yes	No
Fonseca and colleagues, 2009 [38]	Brazil	2005	Hypothetical cohort of 64-year-old postmenopausal women	All	Cost-effectiveness analysis	Model based	Healthcare	Lifetime	Life years saved	Literature based	Expert opinion	On both costs and effects	Yes	Yes
Ginsberg and colleagues, 2012 [27]	Sub-Saharan Africa and South East Asia	2005	Female population at risk, in SearD and AfrE	All	Cost-effectiveness analysis	Model based	Healthcare	100 years	DALYs	Literature based	Secondary data collection	On both costs and effects	Yes	Yes
Salomon and colleagues, 2012 [31]	Mexico	2005	Female population at risk	All	Cost-effectiveness analysis	Model based	Healthcare	100 years	DALYs	Literature based	Secondary data collection	On both costs and effects	Yes	Yes
Pakseresht and colleagues, 2011 [48]	India	2006/2007	103 women with primary breast cancer in a tertiary hospital	All	Cost analysis/cost of illness	Observational	Non-healthcare	2 years	NA	NA	Primary data collection	NA	NA	NA
Yazihan and Yilmaz, 2006 [34]	Turkey	2007	Female population at risk	All	Cost-effectiveness analysis	Other	Healthcare	6 years	DALYs	Secondary data collection	Secondary data collection	None	No	No
Bastani and Kiadaliri, 2012 [49]	Iran	2008	Patients younger than 75 with node-positive breast cancer	All	Cost-utility analysis	Experimental	Healthcare	8 months	QALYs	Primary data collection	Primary data collection	NA	No	NA
Liubao and colleagues, 2009 [39]	China	2008	Model cohort of 1,000 51-year-old operable breast cancer patients	All	Cost-effectiveness analysis	Model based	Healthcare	Lifetime	QALYs	Secondary data collection	Secondary data collection	On both costs and effects	Yes	Yes
Astim, 2011 [36]	Turkey	2010	Female population at risk older than 30	All	Report on costs and effects separately	Model based	Healthcare	10 years	Intermediate outcome measures	Secondary data collection	Literature	Yes	No	No
Zelle and colleagues, 2012 [35]	Ghana	2010	Female population at risk	All	Cost-effectiveness analysis	Model based	Healthcare	100 years	DALYs	Literature based	Primary data collection	On both costs and effects	Yes	Yes
Bai and colleagues, 2012 [42]	China	2012	Model cohort of women aged 51.7, with early stage breast cancer after lumpectomy	1 and 2	Cost-effectiveness analysis	Model based	Healthcare	Lifetime	QALYs	Literature based	Literature/expert opinion	On both costs and effects	Yes	Yes
Arredondo and colleagues, 1995 [43]	Brazil	Not clear	Hypothetical breast cancer case	All	Cost analysis/cost of illness	Observational	Healthcare	NA	NA	NA	Expert opinion	NA	No	No
Boutayeb and colleagues, 2010 [37]	Morocco	Not clear	Early-stage breast cancer patients in Morocco	Not clear	Cost-effectiveness analysis	Observational	Healthcare	1 year	Life years saved	Literature based	Secondary data collection	NA	No	No
Denewer and colleagues, 2010 [26]	Egypt	Not clear	Female population at risk between 25 and 65 years	All	Report on costs and effects separately	Experimental	Healthcare	2 years	Intermediate outcome measures	Primary data collection	Not clear	None	No	No
Guggisberg and colleagues, 2011 [46]	Cameroon	Not clear	Women who underwent FNA in a rural hospital	All	Report on costs and effects separately	Observational	Healthcare	5 weeks	Intermediate outcome measures	Primary data collection	Not clear	NA	No	No
Kobayashi, 1988 [44]	Worldwide	Not clear	NA	NA	Cost analysis/cost of illness	Observational	Healthcare	NA	Intermediate outcome measures	Primary data collection	Primary data collection	NA	NA	NA
Love and colleagues, 2002 [40]	Vietnam and China	Not clear	Premenopausal Vietnamese and Chinese breast cancer patients, considered for surgery	2	Cost-effectiveness analysis	Experimental	Healthcare	15 years	Life years saved	Primary data collection	Not clear	On both costs and effects	No	Yes
Mousavi and colleagues, 2008 [29]	Iran	Not clear	Female population at risk between 35 and 69	All	Report on costs and effects separately	Other	Healthcare	1 year	Life years saved	Expert opinion	Expert opinion	NA	No	No
Nasrinossadat and colleagues, 2011 [47]	Iran	Not clear	51 patients that underwent surgical excision of nonpalpable breast masses	All	Report on costs and effects separately	Observational	Healthcare	3 to 4 years	Intermediate outcome measures	Primary data collection	Not clear	None	No	No
Thomas and colleagues, 1999 [45]	Nigeria	Not clear	Patients who received FNA between 1994 and 1997	All	Report on costs and effects separately	Observational	Patient	NA	Intermediate outcome measures	Primary data collection	Not clear	NA	NA	NA

DALYs, disability-adjusted life years; FNA, fine needle aspiration; NA, not applicable; QALYs, quality-adjusted life years.

**Table 3:** **Interventions compared**, **study objectives**, **and main study conclusions of reviewed articles**

**Authors**	**Interventions compared**	**Study objective**	**Conclusions by authors**
Groot and colleagues, 2006 [28]	Combinations of individual stage I to IV treatment and an extensive mammography screening control program	To assess the cost-effectiveness of breast cancer control that covers various interventions in different settings	Stage I treatment and an extensive screening control program are the most cost-effective interventions
Okonkwo and colleagues, 2008 [30]	Mammography screening, CBE screening among different age groups and in different frequencies	To assess which screening program should be implemented in India	CBE screening in India compares favorably with mammography screening in developed countries
Munshi, 2009 [41]	Several treatment interventions	To present pragmatic cost-saving breast cancer interventions	Intelligent use of knowledge about the disease can help us to exploit new techniques for maximum therapeutic gain with minimal investment
Sarvazyan and colleagues, 2008 [32]	CBE, mammography, ultrasound, magnetic resonance imaging, biopsy, elasticity imaging, tactile imaging	To review the diagnostic accuracy, procedure cost, and cost-effectiveness of currently available techniques for breast screening and diagnosis.	Tactile imaging has the potential to provide cost-effective breast cancer screening and diagnosis
Fonseca and colleagues, 2009 [38]	Anastrozole vs. tamoxifen in the adjuvant setting of early breast cancer	To determine cost-effectiveness of anastrozole, compared with tamoxifen, in the adjuvant treatment of early stage breast cancer in Brazil	Anastrozole is more cost-effective than tamoxifen in the adjuvant setting of early breast cancer
Ginsberg and colleagues, 2012 [27]	Stage 1 to 4 treatment individual, treatment of all stages, biennial mammography screening 50 to 70 vs. null scenario	To determine the cost-effectiveness of 81 interventions to combat breast, cervical and colorectal cancer at different geographic coverage levels, to guide resource allocation decisions in LMICs	For breast cancer, although expensive, mammography screening in combination with treatment of all stages is cost-effective in both regions (I$2,248 to 4,596/DALY). Treating early-stage breast cancer is more cost-effective than treating late-stage disease
Salomon and colleagues, 2012 [31]	Stage 1 to 4 treatment individual, treatment of all stages, screening (annual CBE >25 years + mammography annual >50 years + mammography biennial >40 to 49 years) vs. null scenario	Analyze the cost-effectiveness of 101 intervention strategies directed at nine major clusters of NCDs in Mexico (including breast cancer), to inform decision-makers	Treatment of all stages is cost-effective and treatment of early stages is more cost-effective than late stage treatment. Nationwide screening has an incremental CEA of I$22,000/DALY and is potentially cost-effective
Pakseresht and colleagues, 2011 [48]	NA	To estimate the expenditure audit of women with breast cancer in a tertiary hospital in Delhi	Expenditure on treatment for breast cancer depends on many factors, including the size and stage of the cancer, the woman's age, use of private hospitals and insurance
Szynglarewicz and Matkowski, 2011 [33]	Polish screening program costs vs. other countries	To show preliminary results of the Polish screening program	Population-based mammographic screening conforming the European quality standards is cost-effective even for middle-income countries
Yazihan and Yilmaz, 2006 [34]	Mammography screening in age group 50 to 69 vs. treatment only	To determine the efficiency of resource usage in mammography screenings and the impact on breast cancer stages in Turkey	Mammography screening is economically attractive for Turkey
Bastani and Kiadaliri, 2012 [49]	Docetaxel, doxorubicin and cyclophosphamide (TAC) vs. 5-fluorouracil, doxorubicin, cyclophosphamide (FAC) in node-positive breast cancer patients	To evaluate the cost-utility of TAC and FAC in node-positive breast cancer patients	FAC was a dominant option versus TAC in the short term. In this study, TAC resulted in higher costs and lower QALYs over the study period
Liubao and colleagues, 2009 [39]	AC (doxorubicin/cyclophosphamide) vs. TC (docetaxel/cyclophosphamide)	To estimate the cost-effectiveness of AC (doxorubicin/cyclophosphamide) vs. TC (docetaxel/cyclophosphamide)	TC appears to be more effective and more costly than AC. TC may be viewed as cost-effective using the general WHO threshold
Astim, 2011 [36]	Annual and biennial mammography screening in various age groups (40+, 45+, 50+, 55+, 60+ years) vs. no screening	To evaluate the cost-effectiveness, optimal minimum age and screening interval for a screening program in Turkey	Results of the simulation suggests that women over 40 in Turkey should be screened by mammography biennially
Zelle and colleagues, 2012 [35]	Treatment interventions, biennial mammography and CBE screening interventions, awareness raising interventions, palliative care interventions vs. null scenario	To analyze the cost, effects and cost-effectiveness of breast cancer control interventions in Ghana, and identify the optimal mix of interventions to maximize population health	Both screening by clinical breast examination and mass media awareness raising seem economically attractive interventions ($1,299 and $1,364/DALY). Mammography screening is not cost-effective
Bai and colleagues, 2012 [42]	Radiotherapy vs. no radiotherapy after surgery	To assess the cost-effectiveness of additional radiotherapy for women with early breast cancer after breast-conserving surgery	In health resource-limited settings, the addition of radiotherapy is a very cost-effective strategy (−$420/ QALY) in comparison with no-radio therapy in women with early breast cancer
Arredondo and colleagues, 1995 [43]	Case management costs for infrastructure, human resources, laboratory, hospital stay, drugs, mastectomy, disposable material, curing material	To develop a system for monitoring costs of case management for each disease (breast cancer, cardiac calve disease and enteritis and bronchopneumonia)	Economic analyses hold important information for decision-making
Boutayeb and colleagues, 2010 [37]	Three chemotherapy regimes, AC, AC + taxanes, AC + taxanes + trastuzumab	To evaluate the total cost of chemotherapy in early stage breast cancer	Moroccan health authorities need to devote between US$13.3 to 28.6 million to treat women by chemotherapy every year
Denewer and colleagues, 2010 [26]	CBE-based screening with selective mammography vs. no screening	To evaluate the disease pattern of screen-detected cancers and determine the effectiveness of CBE-based screening	CBE-based screening with selective mammography is feasible, effective and improves the results of breast cancer management in Egypt
Guggisberg and colleagues, 2011 [46]	On-site FNA clinic vs. shipping of specimens	To assess the feasibility of an on-site cytopathology clinic in a rural hospital in Cameroon	Cytopathology (FNA) is a reliable alternative for tissue diagnosis in low-resource settings
Kobayashi, 1988 [44]	Costs and performance of breast echography in different institutions	To analyze the economics and cost performance of breast echography in various institutions	The best cost performance, internationally, can be achieved by mechanical and real-time electronic linear scanners
Love and colleagues, 2002 [40]	Adjuvant oophorectomy and tamoxifen vs. oophorectomy and tamoxifen for recurrence after observation.	To evaluate costs, disease-free and overall survival after surgical oophorectomy and tamoxifen in premenopausal Vietnamese women with operable breast cancer	Vietnamese and Chinese women with hormone receptor-positive operable breast cancer benefit from adjuvant treatment with surgical oophorectomy and tamoxifen
Mousavi and colleagues, 2008 [29]	Mammography screening in age groups 35 to 69 and 50 to 69 and no screening	To decide whether mammography screening should be established in Iran or whether other options are needed	Benefits of other policies than mammography screening need to be explored
Nasrinossadat and colleagues, 2011 [47]	Methylene blue dye injections vs. wire localization	To report experience in marking nonpalpable breast masses by injection of methylene dye	Marking with methylene blue dye is a simple, effective and low-cost method for localization of nonpalpable breast masses
Thomas and colleagues, 1999 [45]	FNA cytology vs. surgical tissue biopsy	To assess the results and limitations of a Nigerian FNA clinic	FNA cytology can help improve the management and cost of care of patients with palpable masses

CEA, cost-effectiveness analysis; CBE, clinical breast examination; DALY**,** disability-adjusted life year; FNA, fine needle aspiration; LMIC, low- and middle-income country; NCD, noncommunicable disease; QALY, quality-adjusted life year; WHO, World Health Organization.

The methodological study characteristics of the reviewed studies are presented in Table 2. The base year of cost data in the included studies was generally not from before year 2000, and could not be identified in eight studies. The majority of studies combined both costs and effects in a single cost-effectiveness estimate (*n* = 13), and the majority of these were based on mathematical models (*n* = 9). Most studies used a healthcare perspective (*n* = 19), and only one study included non-healthcare costs [48]. Studies used a time horizon varying between 5 weeks and the lifetime of the study population. Most reviewed studies used intermediate outcome measures (that is, clinical effects *n* = 8), life years saved (*n* = 6), or disability-adjusted life years (*n* = 5) as their main effectiveness outcome, while quality-adjusted life years were less frequently used (*n* = 3).

### Study quality

Table 4 summarizes the quality of the included studies, as indicated by the percentage score. The quality of all studies ranges from 23 to 86%. Studies by Ginsberg and colleagues, Zelle and colleagues, and Bai and colleagues had the highest total average scores, and these were all modeling studies [27,35,42]. If items were not applicable (NA) for a reviewed paper, the maximum obtainable (domain) score was reduced with 2 points per item.

Studies generally scored poorly on the domain ‘estimation of costs’, at an average 34% of the maximum obtainable score across all studies. The average score for ‘study design’ was 73%, while the quality of the domains ‘estimation of effectiveness’, ‘analysis’, and ‘interpretation of results’ was scores as 70%, 51%, and 68%, respectively.

### Study findings

As described earlier, most studies evaluated breast cancer screening in combination with treatment. Studies in Mexico, Poland, Turkey identified mammography screening as a cost-effective intervention [31,33,34,36], whereas studies in India, Ghana and Egypt found other strategies (such as CBE screening or mass-media awareness raising) to be economically more attractive (Table 3) [26,30,35]. Sarvazyan and colleagues proposed another breast cancer screening option: tactile imaging as an alternative to several other interventions [32].

**Table 4:** Summary of quality assessment and domain scores of reviewed studies

**Authors**			**Scored domains**				**Summary scores**		
		**Study design**	**Effectiveness estimation**	**Cost estimation**	**Analysis**	**Interpretation of results**	**Number of items scored**	**Sum of scores**	**Total average score**
Groot and colleagues, 2006 [28]	Score granted	12	7	6	16	9	29	50	1.72
	% of maximum (domain) score	86%	88%	75%	89%	90%			86%
Okonkwo and colleagues, 2008 [30]	Score granted	12	6	3	16	10	28	47	1.68
	% of maximum (domain) score	86%	100%	38%	100%	100%			84%
Munshi, 2009 [41]	Score granted	7	7	0	1	4	21	19	0.90
	% of maximum (domain) score	50%	70%	0%	50%	40%			45%
Sarvazyan and colleagues, 2008 [32]	Score granted	7	7	0	1	4	21	19	0.90
	% of maximum (domain) score	50%	70%	0%	50%	40%			45%
Fonseca and colleagues, 2009 [38]	Score granted	14	6	1	13	10	28	44	1.57
	% of maximum (domain) score	100%	100%	13%	72%	100%			79%
Ginsberg and colleagues, 2012 [27]	Score granted	12	8	8	18	10	29	52	1.79
	% of maximum (domain) score	86%	100%	75%	89%	100%			90%
Salomon and colleagues, 2012 [31]	Score granted	12	6	5	14	8	29	45	1.55
	% of maximum (domain) score	86%	75%	63%	78%	80%			78%
Pakseresht and colleagues, 2011 [48]	Score granted	7	1	4	3	5	15	20	1.33
	% of maximum (domain) score	88%	50%	50%	75%	63%			67%
Szynglarewicz and Matkowski, 2011 [33]	Score granted	5	3	2	1	5	24	15	0.625
	% of maximum (domain) score	88%	50%	50%	75%	63%			33%
Yazihan and Yilmaz, 2006 [34]	Score granted	12	0	3	2	5	28	22	0.79
	% of maximum (domain) score	86%	0%	38%	13%	50%			40%
Bastani and Kiadaliri, 2012 [49]	Score granted	13	8	4	7	8	25	40	1.6
	% of maximum (domain) score	93%	100%	50%	70%	80%			80%
Liubao and colleagues, 2009 [39]	Score granted	13	7	4	16	10	29	50	1.72
	% of maximum (domain) score	93%	88%	50%	89%	100%			86%
Astim, 2011 [36]	Score granted	9	5	3	8	7	28	32	1.14
	% of maximum (domain) score	64%	63%	38%	50%	70%			57%
Zelle and colleagues, 2012 [35]	Score granted	14	7	7	14	10	29	52	1.79
	% of maximum (domain) score	100%	88%	88%	78%	100%			90%
Bai and colleagues, 2012 [42]	Score granted	13	8	5	18	8	29	52	1.79
	% of maximum (domain) score	93%	100%	63%	100%	80%			90%
Arredondo and colleagues, 1995 [43]	Score granted	10	NA	1	0	7	18	18	1.00
	% of maximum (domain) score	71%	NA	13%	0%	70%			50%
Boutayeb and colleagues, 2010 [37]	Score granted	12	4	4	1	6	25	27	1.08
	% of maximum (domain) score	86%	50%	50%	13%	60%			54%
Denewer and colleagues, 2010 [26]	Score granted	10	4	0	2	5	25	21	0.84
	% of maximum (domain) score	71%	50%	0%	20%	50%			42%
Guggisberg and colleagues, 2011 [46]	Score granted	3	6	2	1	5	25	24	0.96
	% of maximum (domain) score	21%	75%	25%	13%	50%			35%
Kobayashi, 1988 [44]	Score granted	4	4	1	NA	3	19	12	0.63
	% of maximum (domain) score	29%	67%	13%	NA	30%			32%
Love and colleagues, 2002 [40]	Score granted	9	6	1	10	8	27	34	1.26
	% of maximum (domain) score	64%	100%	13%	63%	80%			63%
Mousavi and colleagues, 2008 [29]	Score granted	5	1	0	1	3	22	10	0.45
	% of maximum (domain) score	36%	25%	0%	13%	30%			23%
Nasrinossadat and colleagues, 2011 [47]	Score granted	75	5	0	0	5	25	17	0.68
	% of maximum (domain) score	50%	63%	0%	0%	50%			34%
Thomas and colleagues, 1999 [45]	Score granted	7	4	0	0	6	21	17	0.81
	% of maximum (domain) score	50%	67%	0%	0%	60%			41%
Total average domain score (%)		73%	70%	34%	51%	68%			

Studies evaluating treatment interventions typically favored the novel interventions. Anastrozole was more cost-effective than tamoxifen in a Brazilian study [38], oophorectomy and tamoxifen after recurrence was shown to be favorable in Vietnamese and Chinese patients [40], additional radiotherapy after breast-conserving surgery was very cost-effective in China [42], and chemotherapy consisting of a docetaxel and cyclophosphamide regimen was more attractive compared with an doxorubicin and cyclophosphamide regimen also in Chinese patients [39]. There was only one study with a negative suggestion for the novel and more costly intervention docetaxel, doxorubicin, cyclophosphamide, as compared with the more conventional 5-fluorouracil, doxorubicin, dyclophosphamide regime [49].

Studies that only assessed costs and did not include effectiveness estimates, reported on costs of breast cancer for patient management in Brazil (US$1,646 per patient) [43], and the costs of patient expenditure (US$242 per patient) in India [48].

The three studies evaluating diagnostic interventions demonstrated the economic attractiveness of inexpensive interventions; that is, fine-needle aspiration cytology and methylene blue dye injections [45-47]. These interventions could be especially relevant for diagnosing breast cancer in rural settings and settings with low resources.

## Discussion

This study shows that there is limited economic evidence on breast cancer control in LMICs. Only 24 economic evaluation studies were found in this review, and their quality was generally poor. Furthermore, the study populations were very diverse, as most studies examined different kinds of screening and therapeutic interventions in various age and risk groups. Owing to this poor availability, quality, and comparability, we conclude that the economic evidence base to guide strategies for breast cancer in LMICs is currently insufficient.

Our review raises a few discussion points. First, there is mixed evidence on the economic attractiveness of mammography screening. Studies in Mexico, Poland and Turkey demonstrate the intervention to be cost-effective, whereas studies in India, Ghana, and Egypt suggests that other forms of screening – for example, by CBE – provide more value for money. The evidence base is too small to generalize these findings to other LMICs, and to draw general conclusions. Also, most of the studies evaluating therapeutic interventions seem to favor the more novel – and often more expensive – therapy. These findings may be explained by many reasons, including the higher effectiveness of the novel interventions but possibly also the association between funding sources and pro-industry conclusions [50].

Second, in general, we found that the quality of the reviewed articles was poor. The majority of studies failed to score at least 50% on every domain (‘study design’, ‘estimation of effectiveness’, ‘estimation of costs’, ‘analysis’, and ‘interpretation of results’). These domain scores further show that most emphasis was given to the design of the studies and the interpretation of results, whereas costs, in particular, were poorly evaluated. This calls for better adherence of studies to methodological standards for economic analyses, or the development of such standards specifically for breast cancer research. Future studies could be improved by using a checklist, and through transparent reporting of the items in checklists [25,51].

Third, the current evidence base leaves many LMICs with the difficult task of extrapolating results from other countries. The transferability of economic evaluations across countries is complicated, as clinical practice patterns, healthcare systems, and cultural and ethical practices differ across countries [52,53]. Standardized ways of adopting economic evaluations, with the help of available checklists and guidelines [24,25,51,54-58], may improve this lack of transferability. Alternatively, modeling studies could play an important role in extrapolating results from one context to another. Modeling studies, however, rely on the availability of costing and effectiveness data, and this emphasizes the need for more primary data collection on these aspects in LMICs. With data from such studies, researchers would not have to continue to rely on sensitivity analyses or extrapolating cost estimates from data in HICs. National cancer registries, mortality databases, hospital registries, and accessible publications would be essential for providing such information [59].

Fourth, and closely related, we generally advocate the use of modeling studies in the economic analysis of breast cancer control in LMICs. In addition to their use in the extrapolation of study findings, they generally appeared to be of high quality, are sufficiently flexible to include important methodological characteristics such as adequate time horizon, and seem also appropriate to evaluate a broad array of interventions across different groups.

Fifth, the most adopted type of economic evaluation was cost-effectiveness analysis, using a healthcare perspective and life years saved as the primary outcome. Although cost-effectiveness analyses using a healthcare perspective contribute very important information, productivity losses for patients suffering from breast cancer – and most probably other NCDs – can be substantial [60,61]. So far, there is no methodological consensus on estimating productivity loss and the cost of illness can vary greatly between different costing approaches (for example, human capital approach vs. friction cost approach) and also between gender, age and the type of job of patients [62]. Further research should account for economic and social characteristics of the population under study, and should try to investigate productivity losses. Additionally, life years saved may be a less appropriate outcome when palliative or preventive interventions are investigated, and the use of disability-adjusted or quality-adjusted life years may be more appropriate.

Sixth, there is currently very little economic evidence on less established interventions such as tactile imaging, awareness raising, CBE screening, or preventive and palliative interventions. Economic studies, especially in LMICs, should aim to evaluate these interventions more often (and thereby including broad target populations) as they have the potential to be economically attractive [26,30,32,35].

Finally, guidance in decision-making and recommendations for implementation are generally underemphasized in economic evaluations. By reflecting on the health system characteristics of the particular country and considering them in implementation recommendations, economic evaluations could improve their use in breast cancer policy development.

Our study has a number of limitations. Primarily, the number of articles reviewed is very limited, possibly the result of our search strategy. Besides a possible publication bias – studies with negative outcomes are less likely to be published – we searched only for articles published in English. This may explain the relatively small number of articles found, for instance, from Spanish-speaking regions or from countries where there is less emphasis on publishing research (for example, in Africa). Also, the studies included in our review vastly differed with regard to their methodology, objectives, characteristics, and study populations and hence are difficult to compare. In addition, our quality assessment of the reviewed articles was based on a checklist that gives highest scores to a full reporting of all domains. However, short reports in the form of, for example, editorials may not include all these details but may nevertheless be valid for the goals they serve. Hence, the scores for these studies should be interpreted with caution.

## Conclusions

To conclude, our findings indicate that research on the costs and cost-effectiveness of breast cancer control in LMICs is still in its infancy. The limited evidence base suggests that screening strategies may be economically attractive in LMICs – yet there is very little evidence to provide specific recommendations (on screening by mammography vs. CBE, the frequency of screening, or the target population). These results demonstrate the need for more economic analysis that are uniform, of better quality, cover a comprehensive set of interventions and result in clear policy recommendations.

## Abbreviations

CBE: Clinical breast examination; HIC: High-income country; LMIC: Low- and middle-income country; NCD: Noncommunicable disease.

## Competing interests

The authors declare that they have no competing interests.

## Authors’ contributions

SGZ performed the search strategy, designed the inclusion criteria, reviewed all papers included in the review, developed the evaluation strategy and drafted the manuscript. RMB participated in the design of the study, the selection of relevant articles, the evaluation and classification of articles and contributed to the writing of the manuscript. Both authors reviewed and critically assessed the papers included in this review.
